# Descriptive study of cases of schizophrenia in the Malian population

**DOI:** 10.1186/s12888-021-03422-9

**Published:** 2021-08-20

**Authors:** Souleymane dit Papa Coulibaly, Baba Ba, Pakuy Pierre Mounkoro, Brehima Diakite, Yaya Kassogue, Mamoudou Maiga, Aperou Eloi Dara, Joseph Traoré, Zoua Kamaté, Kadiatou Traoré, Mahamadou Koné, Boubacar Maiga, Zoumana Diarra, Souleymane Coulibaly, Arouna Togora, Youssoufa Maiga, Baba Koumaré

**Affiliations:** 1 Faculty of Medicine and Odontostomatology, University of Sciences, Techniques and Technologies of Bamako (USTTB), 1805, Point G, Bamako, Mali; 2 University Teaching Hospital Point G, Bamako, Mali; 3 Institute for Global Health, Northwestern University, Chicago, IL60611 USA; 4 University Teaching Hospital Gabriel Toure, Bamako, Mali

**Keywords:** Schizophrenia, Socio-demographic, Clinical, Environment, Mali

## Abstract

**Background:**

Schizophrenia is a relatively common disease worldwide with a point prevalence of around 5/1000 in the population. The aim of this present work was to assess the demographic, clinical, familial, and environmental factors associated with schizophrenia in Mali.

**Methods:**

This was a prospective descriptive study on a series of 164 patients aged at least 12 years who came for a follow-up consultation at the psychiatry department of the University Hospital Center (CHU) Point G in Mali between February 2019 and January 2020 for schizophrenia spectrum disorder as defined by DSM-5 diagnostic criteria.

**Results:**

Our results revealed that the male sex was predominant (80.5%). The 25–34 age group was more represented with 44.5%. The place of birth for the majority of our patients was the urban area (52.4%), which also represented the place of the first year of life for the majority of our patients (56.1%). We noted that the unemployed and single people accounted for 56.1 and 61% respectively. More than half of our patients 58.5% reported having reached secondary school level. With the exception of education level, there was a statistically significant difference in the distribution of demographic parameters. Familial schizophrenia cases accounted for 51.7% versus 49.3% for non-familial cases. The different clinical forms were represented by the paranoid form, followed by the undifferentiated form, and the hebephrenic form with respectively 34, 28 and 17.1%. We noted that almost half (48.8%) of patients were born during the cold season. Cannabis use history was not observed in 68.7% of the patients. The proportions of patients with an out-of-school father or an out-of-school mother were 51.2 and 64.2%, respectively.

**Conclusion:**

The onset of schizophrenia in the Malian population has been associated with socio-demographic, clinical, genetic and environmental characteristics.

## Background

Schizophrenia is a relatively common condition worldwide with a point prevalence of approximately 5/1000 in the population [1]. The clinical manifestations of schizophrenia are include delusions, hallucinations and disorganized speech [2]. These clinical features are interpreted as a supernatural fact from the point of view of cultural beliefs [3]. Schizophrenia is a multifactorial disease of unknown causes. However, it is accepted that several factors, in particular environmental and genetic, interact in the development of the disease [4, 5]. According to persistent Africans beliefs, the causes of the disease are mainly of psychosocial and supernatural origin [6]. In sub-Saharan Africa, mental illnesses such as schizophrenia are caused by the invisible world imposing its authority on the visible world [7]. The visible world is everything that lives between heaven and earth, including humans, animals, water, fire, plants, earth, and air. However, the invisible world represents the origin and the final destination of any living being, in this world the geniuses, ancestors as well as the double of each being live under the authority of God. In such consideration, schizophrenia appears to be due to the possession of a person’s body or mind by a jinn, a sorcerer or jealous congener [8]. Diagnostic concepts of schizophrenia are important in the management of the patient at the individual level as well as in the research of risk factors and mechanisms of causality [5].

Many authors have demonstrated the existence of a strong relationship between many factors and the risk of developing schizophrenia, in particular socio-demographic (sex, age, marital status and area of residence), socio-economic (income level), genetic (consanguinity) and environmental (season of birth, cannabis use) [9–14]. It is noteworthy that, most of these investigations have been done in Western countries, where schizophrenia related parameters were well described. Looking at the literature review, data regarding sub-Saharan Africa are lacking, especially Mali.

Malian population in its majority like that of other African countries does not escape the popular belief that schizophrenia is due to the invisible world, that is to say of divine origin. However, the Malian urban population is gradually becoming westernized by its way of life and its access to information. As a result, the families of patients begin to reject the divine origin of the disease and would instead think that the disease is the result of narcotics consumption. In order to determine the phenotypic profile of schizophrenia in Mali and to contribute to world epidemiology data of the disease, we carried out the present study which aims to describe the socio-demographic, clinical, genetic and environmental aspects of the disease.

## Methods

The study was approved by the ethics committee of the Faculty of Medicine and Odontostomatology/Faculty of Pharmacy under the number 2019/63/CE/FMPOS at the University of Sciences, Techniques and Technologies of Bamako (USTTB). Study participants were recruited in the psychiatry department of the University Hospital Center of Point G from February 2019 to January 2020. Each participant received a detailed explanation about the study and was invited to give informed consent. This is a prospective descriptive study involving a series of 164 patients aged at least 15 years followed on an outpatient basis for at least 1 year for a schizophrenia spectrum disorders such as schizophrenia, schizophreniform disorder and schizoaffective disorder according to the diagnostic criteria of DSM-5 (Diagnostic and Statistical Manual of Mental Disorders). Study participants recruitment was based on the DSM-5 criteria. The physician, using an interview guide and pre-established survey form, addressed the patient alone after having established a climate of trust. Then, the physician collected data concerning socio-demographic parameters, including age, gender, professional situation, marital status, level of education, rank in uterine siblings, clinical (age of diagnosis, type, and sub-types of schizophrenia), and environmental (place of birth and life, family’s monthly income, birth season, cannabis use history) and the family history (father-mother consanguinity relationship and the number of relatives with schizophrenia) were collected for all patients. After that, the recorded files were submitted to a second physician for review for the confirmation of the DSM-5 criteria before the patient was considered for the study.

### Operational description of the studied parameters

The age of the participants was determined based on the day, month and year of birth. Participant’s gender was defined on the basis of the observation of a set of characteristics that distinguish male and female. The professional situation was determined according to the regular exercise or not of an activity to earn a living. The educational level of the participants correspond to the higher level achieved by the participant in the institutional education system of the country.

The rank in uterine siblings was defined according to the subsequent chronological order of the births of a mother’s children. Consanguinity characterizes the sharing of a common ancestor. Schizophrenia was defined based of the DSM-5 criteria. The schizophrenia sub-types have been defined by referring to the criteria for the International Classification of Diseases 10th Edition. The concept of cannabis use refers to the positive verbal affirmation of an experience of cannabis use in the last 12 months.

### Statistical analysis

SPSS statistical package version 19.0 Software was used to analyze the data. All categorical variables were presented as proportions. We used the chi-square test to assess the relationship between the socio-demographic, clinical, genetic, environmental variables, and schizophrenia. A *p*-value < 0.05 was considered as statistically significant.

## Results

The present descriptive study concerned 164 Malian patients suffering from schizophrenia. As shown in Table 1, the patients consisted of 132 males (80.5%), 32 females (19.5%) with a mean age of 28.9 ± 12.9; range (15–45 years). The 25–34 age group was more represented with 44.5% compared to other age groups. The place of birth of most of our patients was the urban area with 52.4% versus 39.6 and 7.8% for rural and semi-urban areas, respectively. Regarding professional status, we noted that the unemployed were more frequent with 56.1% compared to active workers (43.3%) and retirees (0.6%). As for marital status, single people were the most frequent with 61% compared to married (31.1%), divorced (5.5%) and widow/widower (2.5%). Overall, 79.3% of patients attended school; however, patients with secondary education level were more represented compared to others (primary, high and unschooled). Patients with average income level was more represented in our study population (58.5%) (Table 1). The socio-demographic analysis showed significant differences in the distribution of the different parameters, except the education level (Table 1).

**Table 1:** Demographic characteristics of patients with schizophrenia

Parameter	N	%	*p*. value
**Gender**	164		< 0.0001
Male	132	80.5	
Female	32	19.5	
**Age**	164		< 0.0001
15–24	36	22.0	
25–34	73	44.5	
35–44	38	23.2	
≥ 45	17	10.4	
**Professional status**	164		< 0.0001
Active worker	71	43.3	
Retired worker	1	0.6	
Unemployed	92	56.1	
**Marital status**	164		< 0.0001
Single	100	61	
Married	51	31.1	
Divorced	9	5.5	
Widower/widow	4	2.4	
**Level of education**	164		0.411
Primary	42	25.6	
Secondary	49	29.9	
High	39	23.8	
Unschooled	34	20.7	
**Place of birth**	164		< 0.0001
Rural	65	39.6	
Urban	86	52.4	
Semi-urban	13	7.9	
**Income level of family**	164		< 0.0001
Low	33	20.1	
Average	96	58.5	
High	35	21.3	

Table 2 displays the clinical characteristics of the study participants. The distribution of familial (51.7%) and non-familial (49.3%) cases of schizophrenia were statistically comparable, *p* = 0.875. We noted that the paranoid form was the most common clinical manifestation of schizophrenia (34.1%) followed by the undifferentiated (28.7%), hebephrenic (17.1%), and other forms whose frequencies varied between 3 and 7.3%. In addition, we observed a statistically significant difference in the distribution of clinical forms of schizophrenia, *p* <  0.0001.

**Table 2:** Clinical features of patients with schizophrenia

Parameter	N	%	***p.*** value
**Types of schizophrenia**	164		0.875
Family cases	81	49.3	
Non-family cases	83	51.7	
**Clinical forms of schizophrenia**	164		< 0.0001
Paranoid	56	34.1	
Hebephrenic	28	17.1	
Catatonic	5	3.0	
Undifferentiated	47	28.7	
Post-schizophrenia depression	8	4.9	
Residual	12	7.3	
Simple	8	4.9	

**Fig. 1:**
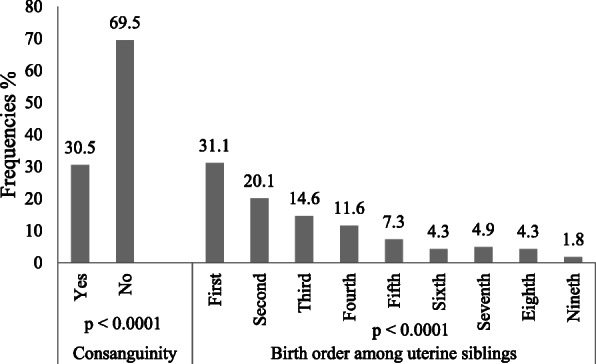
Distribution of schizophrenia patients according to genetic characteristics

When analyzing genetic factors, we found that 30.5% of the study participants had a history of consanguinity with their biological parents (father and mother). Among uterine siblings, the frequency of schizophrenia decreased statistically from firstborn (31.1%) to last (1.8%) in a total order of 9 siblings, as shown in Fig. 1.

In Table 3, analysis of data on environmental factors showed that most schizophrenic patients were born during the cold season (48.8%) followed by the hot season (21.3%), the rainy season (18.9%), and the unspecified season [11]. History of cannabis use was absent in 68.7% of participants versus 32.3% for those who used cannabis, *p* <  0.0001(Table 3).

**Table 3:** Distribution of schizophrenia patients regarding environmental characteristics

Parameter	N	%	***P*** value
**Season of birth**	164		< 0.0001
Cold season	80	48.8	
Hot season	35	21.3	
Rainy season	31	18.9	
Unspecified	18	11	
**Cannabis use**	164		< 0.0001
Yes	53	32.3	
No	111	68.7	

## Discussion

Schizophrenia is one of the major contributors to the global burden of disease. Several factors have been recognized to be involved in the onset of neurodevelopmental disease, including exposure of the fetus viral influenza, rubella, or maternal toxoplasmosis [4, 15–17]. In addition, other studies have demonstrated the effect of the interaction between environmental and genetic factors [18, 19]. As a result, the explanatory model of schizophrenia appears complex like any multifactorial disease [18]. Beyond these assumptions, schizophrenia is recognized as a ubiquitous pathology whose appearance seems to be linked to individual’s age and sex. This is corroborated by the fact that the disease is most often observed in boys between 15 and 24 years old and girls between 25 and 35 years old [20]. The results of the present study showed that schizophrenia was more common in males than in females. Aleman et al. confirmed evidence for gender difference in the risk of developing schizophrenia [21]. Previous investigations have reported evidence that the gender difference in schizophrenia reflects differences both in neurodevelopmental processes and in social effects on risk and disease course [22]. Markham et al. also suggested a protective role of ovarian hormones against the onset of schizophrenia [22]. Gender inequality seems to be confirmed for the Malian population. Indeed, among the patients in hospital psychiatric care in Mali, we find the predominance of the male gender and especially the 23–25 age group [23–25]. The 25 to 34 age group was the most represented in our study. These results were comparable to those reported by Esan et al. who found that the majority of patients diagnosed with schizophrenia in the Southwest Nigerian population were 25–34 years old [11]. Weiser and *al*. and Mounkoro et al. found similar trend with the 25–35 age group [25, 26]. Although a small number of cases of schizophrenia appear after age 40, the majority of cases of schizophrenia occur in adolescence [27]. Mental health disorders like schizophrenia are exacerbated by the lack of structured and working activities for young people. Thus, the unemployed were the most represented in our study sample. This trend has also been observed in some studies carried out by Houngbé et al., Kelede et al. and Marwaha et al., in which significantly high prevalence of schizophrenia were observed among the unemployed in Benin, Ethiopia and the United Kingdom [27–29]. The link between the increased risk of schizophrenia and social disadvantages such as the high unemployment rate has been confirmed by other studies [7]. Generally speaking the lack of work and social support leads to a loss of personality and social independence in many young people, constituting an obstacle to the realization of their dreams. Marital status, in particular single status, has been reported to influence the course of schizophrenia (from onset of the prodrome to subsequent outcome). In our study, single people were more represented in the schizophrenic population. This trend has also been observed in the Ethiopian population, where Kebede et al. found that people who never married had a 3-folds higher risk of developing schizophrenia compared to married people. In addition, they found that the risk of the disease was 6 times higher in individuals who were separated, divorced or widowed [28]. In a review, Messias and *al.* also reported that unmarried people are 4 times more likely to develop schizophrenia than married people [1]. Factors such as relationship avoidance, inability to start or maintain a long-term relationship can expose individual to develop schizophrenia [28]. In the African context, the celibacy of an adult or, even worse, an elderly person is an anomaly. It is perceived as a deviance that profoundly upsets cultural and social models. Celibacy could conceal serious emotional or relational suffering and social isolation. This is particularly true of the elderly who do not have offspring, leading to psychological discomfort. The relational vulnerability appears to affect divorced/separated and single individuals slightly more than couples or widowers. Our study did not reveal any statistical difference between education level of education and schizophrenia. These results were consistent with those of the Ethiopian and Tunisian communities [12, 28]. However, Luo et al. have reported lower risk of schizophrenia among people with additional years of study in the Chinese population [30]. Improving the level of education can prevent schizophrenia. Urban birth is a well-known risk factor for developing schizophrenia and this was supported by certain studies which found that being a male and living in an urban area was an independent risk factor for schizophrenia in Ethiopia and Ireland [28, 31]. In addition, Lundberg et al. concluded that urban birth was associated with the schizophrenia delusions subgroup in the Uganda population [32]. It has also been reported in the population of Copenhagen that the risk of schizophrenia is high in people who lived their first 5 to 15 years in an urban setting [33]. A large meta-analysis including 46,820 cases of psychosis conducted mainly in the European population, revealed that the incidence of schizophrenia measured in terms of population size or density increases significantly in the urban areas compared to rural areas with an estimated risk level of 2.27 [34]. This makes it possible to consider urbanity as a “marker” of the risk of schizophrenia [20]. Our results have shown the same trend with a predominance of schizophrenics having for place of birth and first year of life the urban environment. The risk of schizophrenia increases with the degree of urbanization at birth and this may be linked to traffic, toxins, infections, diet, social class, or selective migration [34]. In the Malian context, our result could also be linked to a bias due to the offer of available psychiatric care. In fact, in Mali, psychiatric care is available in a single university hospital center in Bamako and four other outpatient units located in the administrative regions. The capacity and the resources allocated to these units are very limited. As a result, these ambulatory care units can only ensure continuity of patient care. The country has 0.05 psychiatrists for 1000,000 inhabitants. The frequencies of family (*N* = 83) and sporadic (*N* = 81) cases were statistically similar in our study sample. The same trend was observed in the Afrikaner population in South Africa by Van der Merwe et al., *N* = 149 versus *N* = 130 [10]. In the contrast, most cases of schizophrenia in the Taiwanese and Chinese populations were sporadic [35, 36]. The distribution of schizophrenia types may depend on the population studied, genetic background and environmental factors. The paranoid form was the most common form in our sample. Our results are consistent with those obtained by Campbell et al. [37]. In addition, a study by Stomp and *al.* using the DSM-IV and ICD-10 criteria showed a high rate of paranoid form and a low rate for hebephrenic and catatonic forms in the Austria population of Vienna [38]. On the other hand, undifferentiated forms were very frequent in the Tunisian population followed by Paranoid forms [39]. The first-born siblings were the most affected by schizophrenia in our study (31.1% of cases). Although, several studies have shown the predominance in the first-born, some authors recommend caution in suggesting a causal link [1, 40]. We found a consanguinity rate of 30.5% in schizophrenic patients. In Egypt, Mansour et al. reported a rate of 46.6% in the Nile Delta region [41]. McClain et al. considered inbreeding as an age-dependent risk factor for schizophrenia [13]. Bener et al. also showed in the Qatari population that parental consanguinity was high in schizophrenic patients (41.3%) than in non-schizophrenia controls (28.7%) [42]. However, inbreeding has not been associated with schizophrenia in the highly consanguineous Sudanese community [43]. The association of inbreeding with schizophrenia raises the possibility of general physiological decline and recessive genetic risk factors [44]. In the African context, inbreeding is more common among lower socioeconomic groups, which in turn can lead to a high prevalence of socio-cultural and environmental risk factors for schizophrenia, such as consanguineous marriages, obstetric complications, or exposure to putative causative infectious agents. Consanguineous marriage is allowed among the Malian population, but its prohibition in terms of likely public health benefits is debated in every family. Patients born during the cool season were the most represented with 48.8% of cases and 21.3% were born in the hot season and 18.9% during the rainy season. The relationship between birth in rainy period and schizophrenia was demonstrated in previous works [45, 46]. Several authors support this trend and several hypotheses have been put forward to explain the high frequency of this disease in the rainy season [44, 47, 48]. Among them, exposure to infectious agents, in particular the influenza virus, which is the best documented [26]. The notion of cannabis use was found in 32.3% of our schizophrenic patients and this frequency was higher than that observed in the Tunisian population (6.4%). Schizophrenia and cannabis use seems to have a close relationship. The role of tetrahydrocannabinol (THC) in the onset of psychosis and schizophrenia in the population at risk has already been suspected [49]. Our results also showed a strong representation of schizophrenic patients whose biological parents were unschooled. Cao et al. found that parental education level and childbearing age are associated with an increased risk of schizophrenia in a Chinese population [50].

## Conclusions

The present study assessed the risk factors associated with schizophrenia in a sample of the Malian population. Our results showed a strong representation of familial cases of schizophrenia. In addition, this study supports knowledge of the complexity of environmental factors in schizophrenia.

## Data Availability

The datasets generated and/or analyzed in this study are available from the corresponding author upon reasonable request and with the permission of FMPOS Ethics Committee.
